# Model of End-Stage Liver Disease (MELD) Score as a Predictor of In-Hospital Mortality in Patients with COVID-19: A Novel Approach to a Classic Scoring System

**DOI:** 10.7759/cureus.15179

**Published:** 2021-05-22

**Authors:** Peter M Stawinski, Karolina N Dziadkowiec, Baher Al-Abbasi, Laura Suarez, Larnelle Simms, Nakeya Dewaswala, Pedro Torres, Ayat Al Rubaye, Jesus Pino, Akiva Marcus

**Affiliations:** 1 Internal Medicine, University of Miami JFK Medical Center, Atlantis, USA; 2 Cardiology, University of Miami JFK Medical Center, Atlantis, USA; 3 Gastroenterology and Hepatology, University of Miami JFK Medical Center, Atlantis, USA

**Keywords:** covid-19, mortality, meld, infection, inflammation

## Abstract

Background

Limited data is available for reliable and accurate predictors of in-hospital mortality in patients diagnosed with COVID-19.

Methods

This scientific study is a retrospective cohort study of patients without a known history of liver diseases who were hospitalized with COVID-19 viral infection. Patients were stratified into low score groups (Model of End-Stage Liver Disease [MELD] score <10) and high score groups (MELD ≥10). Clinical outcomes were evaluated, including in-hospital mortality, hospital length of stay, and intensive care unit length of stay (ICU LOS).

Results

Our cohort of 186 COVID-19 positive patients included 88 (47%) women with a mean age of 60 years in the low score group and mean age of 73 years in the high score group. Patients in the high score group were older in age (p<0.0001) and more likely to have history of diabetes mellitus (p=0.0020), stage 3 chronic kidney disease (CKD) (p=0.0013), hypertension (p<0.0001), stroke/transient ischemic attack (TIA) (p=0.0163), asthma (p=0.0356), dementia (p<0.0001), and chronic heart failure (p=0.0055). The in-hospital mortality or discharge to hospice rate was significantly higher in the high-score group as opposed to the low-score group (p=0.0014). Conversely, there was no significant difference among both groups in the hospital length of stay (LOS) and ICU LOS (p=0.6929 and p=0.7689, respectively).

Conclusion

Patients hospitalized with COVID-19 infection and found to have a MELD score greater than or equal to 10 were found to have a higher mortality as compared to their counterparts. Conversely a low MELD score is a very strong indicator of a more favorable prognosis, indicating hospital survival. We propose using the MELD score as an adjunct for risk stratifying patients diagnosed with COVID-19 without prior history of liver dysfunction.

## Introduction

To date, cases of severe acute respiratory coronavirus 2 (SARS-CoV-2), responsible for causing the illness referred to as coronavirus disease 2019 (COVID-19), continue to pose a real and urgent threat to global health. During this public health crisis, when measures have been taken to halt the viral spread, trends and predictors of disease control are vital to help create an understanding of its trajectory [1]. To help reduce the burden on the healthcare system while also efficiently providing the best in patient care, there is a continuous necessity for reliable prognostic indicators for the disease [2]. There is evidence of COVID-19 indirectly or directly affecting the digestive and hepatobiliary systems through a viral inflammatory response [3]. The proposed mechanism suggests that the accumulation of inflammatory factors and cytokines results in an overwhelming viremic response and injury to the gastrointestinal system [3-5].

The MELD (Model of End-Stage Liver Disease) score has been well validated as a severity index in patients with end-stage liver disease and can be used to predict short-term mortality [6]. We extrapolate this relationship further between COVID-19 and the applicability of the MELD score as a useful tool for predicting mortality in patients with COVID-19 without known liver disease. We chose to explore the MELD score in COVID-19 positive patients as it is a dynamic model, based on the multiple parameters, including how effectively the liver excretes bile, how well the liver can produce clotting factors, and the current function of the kidney [5]. This study aims to evaluate the potential for the MELD score - in the first 24 hours of admission - in predicting in-hospital mortality, as well as secondary outcomes of hospital length of stay and ICU length of stay.

## Materials and methods

Study design and participants

This project was a single-centered retrospective observational study performed at JFK Medical Center, Atlantis, Florida, USA. We retrospectively analyzed patients diagnosed with COVID-19 between March 2020 and July 2020, who were diagnosed COVID-19 positive by nasopharyngeal swab using the reverse transcription polymerase chain reaction (RT-PCR) [7]. The patients' electronic health records were analyzed on hospital admission and during the patients’ hospital stay by the study team [7].

Patients included in the study were age 18 years and older and stratified into two groups based on their MELD score in the first 24 hours of admission (low-score vs. high-score) [7]. High score is defined as MELD score ≥ 10, while the low score is defined as < 10. The cut-off point of 10 in MELD score is known to be associated with a greater than 6% three-month mortality in end-stage liver disease [8]. The equation used to calculate the MELD score included: 

MELD = 11.2 x ln(international normalized rate [INR]) + 3.78 x ln(bilirubin, in mg/dL) + 9.57 x ln(creatinine, in mg/dL) + 6.43 [9].

Any value less than 1 is given a value of 1 (i.e., if bilirubin is 0.8, a value of 1.0 is used) to prevent subtraction from any of the three factors, since the natural logarithm of a positive number below 1 (greater than 0 and less than 1) yields a negative value [10]. If the patient has been dialyzed twice within the last seven days, then the value for serum creatinine used should be 4.0 mg/dL [10]. 

All-cause in-hospital mortality was defined as death during the index hospitalization, or it’s surrogate discharge to hospice.

Inclusion criteria

Patients age 18 years and older who were hospitalized with a confirmed positive diagnosis of COVID-19 who had INR, bilirubin, and creatinine collected in the first 24 hours of admission [7].

Exclusion criteria

Patients with known liver disease or for whom INR, bilirubin, and creatinine were not collected in the first 24 hours of hospital admission were also excluded, and asymptomatic patients that did not require hospitalization [7]. Patients who were on warfarin or direct-acting oral anticoagulants (DOACs) were also excluded. 

Data collection

The patients’ hospital record was carefully reviewed by the study team. Patient data including laboratory examinations, medical history, comorbid conditions, complications, demographics, treatments initiated, and outcomes were collected and carefully analyzed. 

Outcomes

The evaluated primary outcome is all-cause in-hospital mortality. Secondary outcomes included: hospital length of stay and ICU length of stay.

Statistical analysis

The JMP program Version 14.0.0 (SAS Institute, Cary, North Carolina, USA) was used for the statistical analysis portion. Continuous variables were expressed as means with standard deviation (±SD). Using ANOVA (F statistic), a comparison of means (baseline characteristics, and predictors) was completed. Categorical variables were compared using a Chi-square test. Significant results reflected a value of p-values < 0.05.

## Results

**Figure 1:**
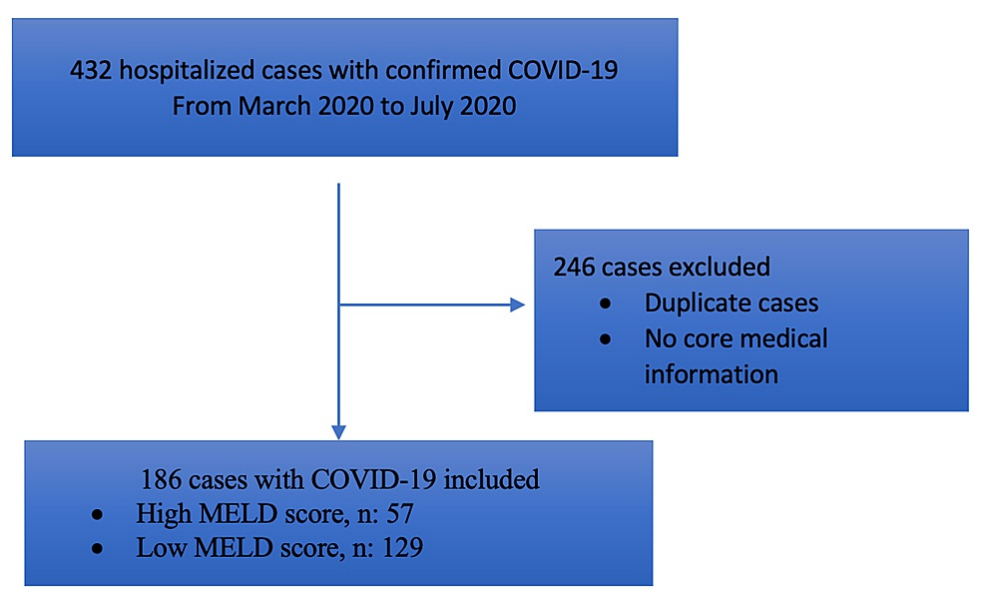
Flowchart for Participants' Selection MELD: Model of End-Stage Liver Disease

We used a total of 432 patient cases with a confirmed positive COVID-19 test result who were screened between March 2020 and July 2020 at a tertiary cardiovascular center [7]. Participant selection is shown in Figure 1. Cases without available core medical information were excluded. A total of 186 hospitalized patients with COVID-19 were included in the final analysis. This study cohort of 186 patients included 88/186 (47%) women with a mean age of 64±17 years.

Comparison of baseline characteristics between high MELD score and low MELD score groups

As demonstrated in Table 1, patients in the high score group were older in age (p<0.0001) and were found to have a history of diabetes mellitus (p=0.0020), stage 3 chronic kidney disease (CKD) (p=0.0013), hypertension (p<0.0001), stroke/TIA (p=0.0163), asthma (p=0.0356), dementia (p<0.0001), and chronic heart failure (p=0.0055). While, there was no significant difference in malignancy, coronary artery disease (CAD), chronic obstructive lung disease (COPD), or peripheral vascular disease (PVD) between the two groups (p=NS [not significant]). Furthermore, patients with a high MELD score on hospital admission were more likely to have several laboratory abnormalities such as an elevated creatinine (p=<0.0001), blood urea nitrogen (p=<0.0001), lactic acid (p=0.0005), total bilirubin (p=<0.0001), alkaline phosphatase (p=0.0299), INR (p=<0.0001), and positive troponin (p=<0.0001), but a low albumin (p=0.0013) and hemoglobin (p=0.0008).

**Table 1:** Baseline characteristics of patients with COVID-19 stratified based on MELD score. * Statistically significant; ^+^ Standard deviation; ^‡^ Asparate aminotransferase; ^†^ Alanine aminotransferase;^ §^ International normalized ratio; ^¥ ^Positive troponin, >0.0012; MELD: Model of End-Stage Liver Disease

N=186	MELD score groups		p-value
Characteristics	High-Score Group N: 57	Low-Score Group N: 129	
Age, years ± SD^+^	73 ± 2	60 ± 1	<0.0001*
Gender, female (%)	24 (42%)	64 (50%)	0.3445
Race, Caucasians (%)	38 (67%)	71 (55%)	0.1200
Ethnicity, non-Hispanic (%)	51 (89%)	98 (76%)	0.0334*
Body mass index, kg/m^2 ^± SD^+^	28.2 ± 1	29.7 ± 0.7	0.2240
Diabetes mellitus (%)	25 (44%)	28 (22%)	0.0020*
Hypertension (%)	42 (74%)	55 (43%)	<0.0001*
Chronic kidney disease (%)	6 (11%)	1 (1%)	0.0013*
Malignancy, localized (%)	5 (9%)	19 (15%)	0.2639
Stroke/TIA (%)	6 (11%)	3 (2%)	0.0163*
Coronary artery disease (%)	9 (16%)	10 (8%)	0.0952
Chronic obstructive lung disease (%)	2 (4%)	4 (3%)	0.8846
Asthma (%)	1 (2%)	14 (11%)	0.0356*
Dementia (%)	19 (33%)	10 (8%)	<0.0001*
Chronic Heart Failure (%)	7 (12%)	3 (2%)	0.0055*
Peripheral vascular disease (%)	4 (7%)	3 (2%)	0.1211
Laboratory data			
Absolute lymphocyte count, (10^3^cells/μL) ± SD^+^	1.2 ± 0.5	1.6 ± 0.3	0.4507
Hemoglobin, g/dL ± SD^+^	12.4 ± 0.3	13.5 ± 0.2	0.0008*
Platelets, (× 10^9^/L) ± SD^+^	246 ± 16	256 ± 11	0.6417
Sodium, mEq/L ± SD^+^	138 ± 0.7	135 ± 0.5	0.0021*
Blood urea nitrogen, mg/dL ± SD^+^	46 ± 2.7	16 ± 1.8	<0.0001*
Creatinine, mg/dL ± SD^+^	1.9 ± 0.1	0.8 ± 0.1	<0.0001*
Lactic acid >2 mmol/L (%)	3.1 ± 0.4	1.4 ± 0.3	0.0005*
Troponin, positive^¥^ (%)	33 (58%)	27 (21%)	<0.0001*
Total bilirubin, mg/dL ± SD^+^	0.8 ± 0.1	0.5 ± 0.1	<0.0001*
Albumin g/L ± SD^+^	3.6 ± 0.1	3.8 ± 0.1	0.0013*
AST^‡^ units/L ± SD^+^	194 ± 72	64 ± 48	0.1363
ALT^†^ units/L ± SD^+^	144 ± 59	52 ± 39	0.1998
Alkaline phosphatase, units/L ± SD^+^	110 ± 7	92 ± 4.7	0.0299*
INR§ ± SD^+^	1.5 ± 0.1	1.1 ± 0.1	<0.0001*

Comparison of baseline characteristics between survivor and non-survivor groups

As demonstrated in Table 2, the mean age of non-survivors were a decade older (72.7 vs. 61.6 years of age) (p=0.0002). These patients in the non-survivor group had a history of hypertension (75%, p=0.0005), chronic kidney disease (9%, p=0.0336), dementia (36%, p=0.0001), and chronic heart failure (11%, p=0.0439). While there was no significant difference in non-survivors versus survivor group in gender (p=0.5300), race (p=0.3930), body mass index (BMI) (p=0.7569), diabetes mellitus (p=1857), malignancy (0.1682), stroke/TIA (p=0.4837), coronary artery disease (CAD) (p=0.3911), chronic obstructive lung disease (COPD) (p=0.6822), asthma (p=0.3265), and peripheral vascular disease (PVD) (p=0.5521). Furthermore, patients in the non-survival group showed to have additional biochemical abnormalities such as an elevated sodium (p=0.0459), blood urea nitrogen (BUN) (p=0.0001), creatinine (p=0.0011), lactic acid >2 mol/L (p=0.0001), total bilirubin (p=0.0203), and INR (p=0.0047). 

**Table 2:** Baseline characteristics of patients with COVID-19 stratified based on mortality * Statistically significant; ^+^ Standard deviation; ^‡^ Asparate aminotransferase; ^†^ Alanine aminotransferase; ^§^ International normalized ratio; ^¥^ Positive troponin, >0.0012;^ f^ Model End-Stage Liver Disease

N= 186	Non- survival N: 44	Survival N: 142	p-value
Characteristics			
Age, years ± SD^+^	72.7 ± 2.5	61.6 ± 1.4	0.0002*
Gender, female (%)	19 (43%)	69 (49%)	0.5300
Race, Caucasians (%)	29 (66%)	80 (56%)	0.3930
Body mass index, kg/m^2 ^± SD^+^	29 ± 1.2	29 ± 0.7	0.7569
Diabetes mellitus (%)	16 (36%)	37 (26%)	0.1857
Hypertension (%)	33 (75%)	64 (45%)	0.0005*
Chronic kidney disease stage (%)	4 (9%)	3 (2%)	0.0336*
Malignancy (%)	3 (7%)	21 (15%)	0.1682
Stroke/TIA (%)	3 (7%)	6 (4%)	0.4837
Coronary artery disease (%)	6 (14%)	13 (9%)	0.3911
Chronic obstructive lung disease (%)	1 (2%)	5 (4%)	0.6822
Asthma (%)	2 (5%)	13 (9%)	0.3265
Dementia (%)	16 (36%)	13 (9%)	<0.0001*
Chronic Heart Failure (%)	5 (11%)	5 (4%)	0.0439*
Peripheral vascular disease (%)	1 (2%)	6 (4%)	0.5521
Laboratory data			
Absolute lymphocyte count ± SD^+^	1.2 ± 0.6	1.6 ± 0.3	0.5687
Hemoglobin, g/dL ± SD^+^	13 ± 0.3	13 ± 0.2	0.8460
Platelets, (× 10^9^/L) ± SD^+^	252 ± 19	253 ± 11	0.9812
Sodium, mEq/L ± SD^+^	138 ± 0.8	136 ± 0.5	0.0459*
Blood urea nitrogen, mg/dL ± SD^+^	38.4 ± 3.5	21 ± 2	<0.0001*
Creatinine, mg/dL ± SD^+^	1.6 ± 0.1	1 ± 0.1	0.0011*
Lactic acid >2 mmol/L (%)	3.6 ± 0.4	1.3 ± 0.3	<0.0001*
Troponin, positive^¥^ (%)	28 (63%)	32 (23%)	<0.0001*
Total bilirubin, mg/dL ± SD^+^	1.1 ± 0.04	1 ± 0.02	0.0203*
Albumin g/L ± SD^+^	3.6 ± 0.1	3.8 ± 0.03	0.0011
AST units/L ± SD^+^	238 ± 82	62 ± 46	0.0629
ALT units/L ± SD^+^	185 ± 67	48 ± 37	0.0768
Alkaline phosphatase, units/L ± SD^+^	105 ± 8	96 ± 5	0.3202
INR§ ± SD^+^	1.4 ± 0.01	1.2 ± 0.04	0.0047*
MELD^f^ score, low(%)	22 (17%)	107 (83%)	0.0014*
MELD^f^ score, high(%)	22 (39%)	35 (61%)	0.0014*

Outcomes

Patients with a high MELD score on admission had a higher all-cause in-hospital mortality rate of 39% vs. 17% (p=0.0014) in comparison to the low score group. Conversely, there was no significant difference between low- and high-score groups in the secondary outcomes of hospital LOS and ICU LOS (p=0.6929 and p=0.7689, respectively) (Table 3).

**Table 3:** Outcomes of patients with COVID-19 stratified by MELD score * Statistically significant; ^+^ Standard deviation; ^‡^ Intensive care unit; MELD: Model of End-Stage Liver Disease

N= 186	MELD score groups		p-value
	High-Score Group N: 57	Low-Score Group N: 129	
Primary outcome			
In-hospital mortality or discharge to hospice care (%)	22 (39%)	22 (17%)	0.0014*
Secondary outcomes			
Hospital length of stay, days ± SD^+^	9.1 ± 1.5	8.4 ± 0.9	0.6929
ICU‡ length of stay, days ± SD^+^	9.8 ± 2.4	10.7 ± 2	0.7689

Predictive values of MELD score for in-hospital mortality in patients with COVID-19

As demonstrated in Table 4, a low MELD score in the first 24 hours of admission had a negative predictive value of 82.95% with a 95% CI (78.1% - 86.9%) and a positive predictive value of 38.6% with 95% CI ( 29.39% - 48.70%) for all-cause in-hospital mortality (i.e., 82.95% is the probability that a patient with a low MELD score on admission discharged home alive and 38.6% is the probability that a patient with an high MELD score on admission died during the index hospitalization).

**Table 4:** MELD score as a predictor of in-hospital mortality in patients with COVID-19 *The prevalence of all-cause in-hospital mortality for the study population. Sensitivity, specificity, disease (mortality) prevalence, positive and negative predictive value and accuracy are expressed as percentages. Confidence intervals for sensitivity, specificity and accuracy are "exact" Clopper-Pearson confidence intervals. Confidence intervals for the likelihood ratios are calculated using the "Log method" as shown on page 109 of Altman et al. 2000 [11]. Confidence intervals for the predictive values are the standard logit confidence intervals given by Mercaldo et al. 2007 [12]. MELD: Model of End-Stage Liver Disease

Statistic	Value	95% CI
Sensitivity	50%	34.56% to 65.44%
Specificity	75%	67.42% to 82.19%
Positive Likelihood Ratio	2.03	1.34 to 3.06
Negative Likelihood Ratio	0.66	0.49 to 0.9
Disease (mortality) prevalence	23.66% (mortality)*	
Positive Predictive Value	38.6%	29.39% to 48.7%
Negative Predictive Value	82.95%	78.1% to 86.9%
Accuracy	69.35%	62.19% to 75.89%

## Discussion

Our study demonstrates that the MELD score has the potential to predict all-cause in-hospital mortality in patients with COVID-19 using a cut-off score of 10 (given it is associated with a greater than 6% three-month mortality in end-stage liver disease) [8]. In patients with COVID-19, abnormal liver function and high MELD score may result from direct viral damage, immune-mediated inflammation, and hypoxia-reperfusion dysfunction [13].

Some studies have found that bile duct epithelial cells and hepatocytes may also express angiotensin-converting enzyme 2 (ACE2) receptors and since it is well known that SARS-CoV-2 gain access to the cells through ACE2 receptors, this suggest that SARS-CoV-2 infection might also cause direct damage to bile duct epithelial cells and hepatocytes [14, 15].

In addition, COVID-19 positive patients were found to have sudden deterioration, resulting in multi-organ failure. Most literature documented that the occurrence of multi-organ failure is mainly related to the sudden initiation of an inflammatory “storm” in the critically ill COVID-19 patients, increased levels of acute inflammatory markers suggesting end-organ damage including but not limited to the liver and kidney and which incurs a higher risk of mortality [16, 17]. 

Lastly, hypoxia and shock induced by COVID-19-related complications (such as respiratory distress syndrome, systemic inflammatory response syndrome, septic shock, and multi-organ failure) may also cause hepatic ischemia and hypoxia-reperfusion dysfunction leading to worsening of the liver function [17].

Describing the individual parameters of the MELD score in greater details, serum bilirubin can be further increased in situations with hemolysis, blood transfusion, and variability of bilirubin metabolism, factors which were not present in our patient population. Numerous studies showed patients with severe COVID‐19 displayed higher bilirubin levels compared to those with milder forms [18-22]. Secondly, serum creatinine was elevated in the high score cut-off group, likely in the setting of demand ischemia, mentioned earlier, which leads to greater organ dysfunction contributing to mortality. Lastly, INR, having the largest weight in the MELD score, noted elevated in severe COVID-19 infection, as the latest also affects liver function, causing derangements in the coagulation cascade leading to abnormal INR [18]. 

At the time of this writing, there is no data that correlates the use of the MELD score and extrapolates it to predict in-hospital mortality or survival in patients with COVID-19 infection. COVID-19 continues to be a challenging viral disease to manage, that continues to develop and spread across the globe. Identifying patients that are at higher risk with an elevated MELD score in the first 24 hours of hospitalization, can increase the opportunity to provide these patients with advance therapy and likely improve prognosis and outcomes, conversely identifying patients with a low risk with a low MELD score can help to expedite discharge and limit the growing burden on health care system. Therefore, a prognostic scoring system, such as the MELD can help mitigate this dilemma. The advantage of the MELD is several-fold: with only a few baseline laboratory parameters needed to calculate the score, it translates into an important role in predicting COVID-19 mortality.

Limitations

This study is retrospective, which can potentially pose a lot of challenges and biases. Secondly, the mean age of the study population was 64 years of age, this suggests an older population with multiple comorbidities which can misrepresent younger and asymptomatic or mildly symptomatic population. Lastly, our data is limited to its immediate geographical area in South Florida, which has a large elderly community. These patients may or may not have better access to medical care, which maybe misrepresentative of the general population.

## Conclusions

COVID-19-associated liver dysfunction may be considered as the result of primary or secondary liver damage caused mainly by several factors, such as the direct injury, systemic inflammatory response, respiratory distress syndrome-induced hypoxia, and multiple organ failure. In addition patients with more comorbidities as per the Charlson comorbidity index, may mean a higher mortality in COVID patients. The components of the MELD score are essentially a subcomponent of the Charlson comorbidity index. Calculating a MELD score requires only a few parameters, is less time consuming, and yields reliable prognostication. 

This information will hopefully contribute to current literature and clinical knowledge for physicians to help manage COVID-19 and may facilitate conversation in the medical setting about risk stratification, strategies for in-hospital management of patients with COVID-19, and allocation of healthcare resources during the COVID-19 pandemic.
